# Microbial transformation of secondary bile acids: roles in gut ecology and autoimmune diseases

**DOI:** 10.3389/fimmu.2026.1769792

**Published:** 2026-03-10

**Authors:** Lulu Ren, Mengxin Li, Linping Wu, Zhiming Lin, Anping Chen

**Affiliations:** 1 Department of Rheumatology and Immunology, Qinzhou First People’s Hospital, Qinzhou, China; 2 State Key Laboratory of Immune Response and Immunotherapy, Guangzhou Institutes of Biomedicine and Health, Chinese Academy of Sciences, Guangzhou, China; 3 China-New Zealand Joint Laboratory of Biomedicine and Health, Laboratory of Computational Biomedicine, Institute of Drug Discovery, Guangzhou, China; 4 Department of Rheumatology, Third Affiliated Hospital of Sun Yat-Sen University, Guangzhou, China

**Keywords:** autoimmune diseases, gut microbiota, immunomodulation, intestinal ecology, secondary bile acids

## Abstract

Secondary bile acids (SBAs) attracted interest due to their regulatory functions in gut microbial ecology and immune responses. These intricate microbial transformations decisively shape the biochemical properties of SBAs. Recent advancements in artificial intelligence and mass spectrometry technologies have substantially expanded our understanding of the diversity within the SBAs pool. To date, hundreds of SBAs, a minor portion of the natural SBA repertoire, have been identified, alongside the prediction of tens of thousands of associated enzymes. Integrative multi-omics studies have further substantiated the role of SBAs in the pathogenesis of autoimmune diseases. This review synthesizes current knowledge on the microbial modification of bile acids, their effects on gut microbial ecology and immune function, with a particular emphasis on autoimmune disorders. Collectively, these findings highlight SBAs as critical regulators of gut microbiota and immune system homeostasis, with their functions intricately linked to their molecular structures.

## 1 Introduction

The gut microbiota, comprising thousands of microbial taxa, is a key regulator of host health, however, there is a critical gap in our knowledge of how microbiota influences host at the systemic level (1). Within constrained intestinal niches, these communities engage in intricate competitive and cooperative interactions that help shield the host epithelium from exogenous pathogens (2). Research across disciplines has progressively unraveled deep, bidirectional connections between the microbiota and host physiology (3).

Although microbe-host interactions can exhibit stochastic elements (4), the microbiota at a macroscopic level is largely maintained in a dynamic equilibrium in vivo (5). Metabolic exchanges among microbes are fundamental to modulating these fluctuations and sustaining homeostasis (5). In healthy states, direct microbial translocation across the intestinal barrier is limited; consequently, microbial metabolites have emerged as primary signaling mediators through which the microbiota influences host processes (6). Among these, the microbial transformation of primary bile acids (PBAs) into secondary bile acids (SBAs) is a cornerstone of gut ecological stability (7). This conversion provides commensal bacteria with a competitive edge by enabling energy harvest from conjugated bile acids while increasing resistance to SBA toxicity (8, 9). Once absorbed, SBAs enter the systemic bile acid pool and act as signaling ligands via host receptors, directly shaping immune cell differentiation and function. These receptor-driven interactions are vital for immune homeostasis and are increasingly linked to immune-mediated diseases (10).

Autoimmune diseases (ADs), marked by loss of self-tolerance and chronic inflammation, represent a major clinical challenge. Advances in next-generation sequencing (NGS) and multi-omics approaches have consistently connected gut dysbiosis and altered bile acid profiles with AD manifestations (11). Although the repertoire and diversity of identified SBAs have substantially expanded, the causative mechanisms of pathogenesis and treatment warrant further investigation (12, 13). This review aims to synthesize current understanding of the microbial biotransformation of SBAs and their interplay with the host immune system. We will focus on elucidating SBA-mediated immunomodulatory mechanisms and discuss emerging therapeutic strategies that target SBA signaling to dampen autoimmune responses and alter disease trajectories.

## 2 Enterohepatic circulation of bile acids

The process of synthesis, transport and metabolism of PBAs has been extensively characterized (14). In humans, the PBAs comprise cholic acid (CA) and chenodeoxycholic acid (CDCA). Hepatocytes generate PBAs from cholesterol through the classical pathway (also named neutral pathway) and the alternative pathway (also named acidic pathway). The classical pathway is responsible for the majority of PBAs production, whereas the alternative pathway predominantly synthesizes CDCA, which constitutes approximately 10% of the total PBAs pool, and CA and CDCA are produced in nearly equivalent proportions (15). Notably, in rodents, PBAs also include β-muricholic acid and minor quantities of ursodeoxycholic acid (UDCA), the C7-epimer of CDCA (16). Both age and sex exert significant effects on the composition of the bile acid pool and the rates of bile acid synthesis in humans and rodents alike (17, 18). Rodent knockout models designed to mimic the human bile acid profile demonstrate that rodents exhibit limited adaptability to hydrophobic bile acid profiles, resulting in physiological trade-offs. PBAs undergo conjugation with amino acids to enhance their solubility; glycine conjugation predominates in humans, whereas taurine conjugation is the principal form in mice (19). These conjugated PBAs are subsequently transported to the gallbladder via the bile salt export pump (BSEP), storage as bile (20, 21).

**Figure 1:**
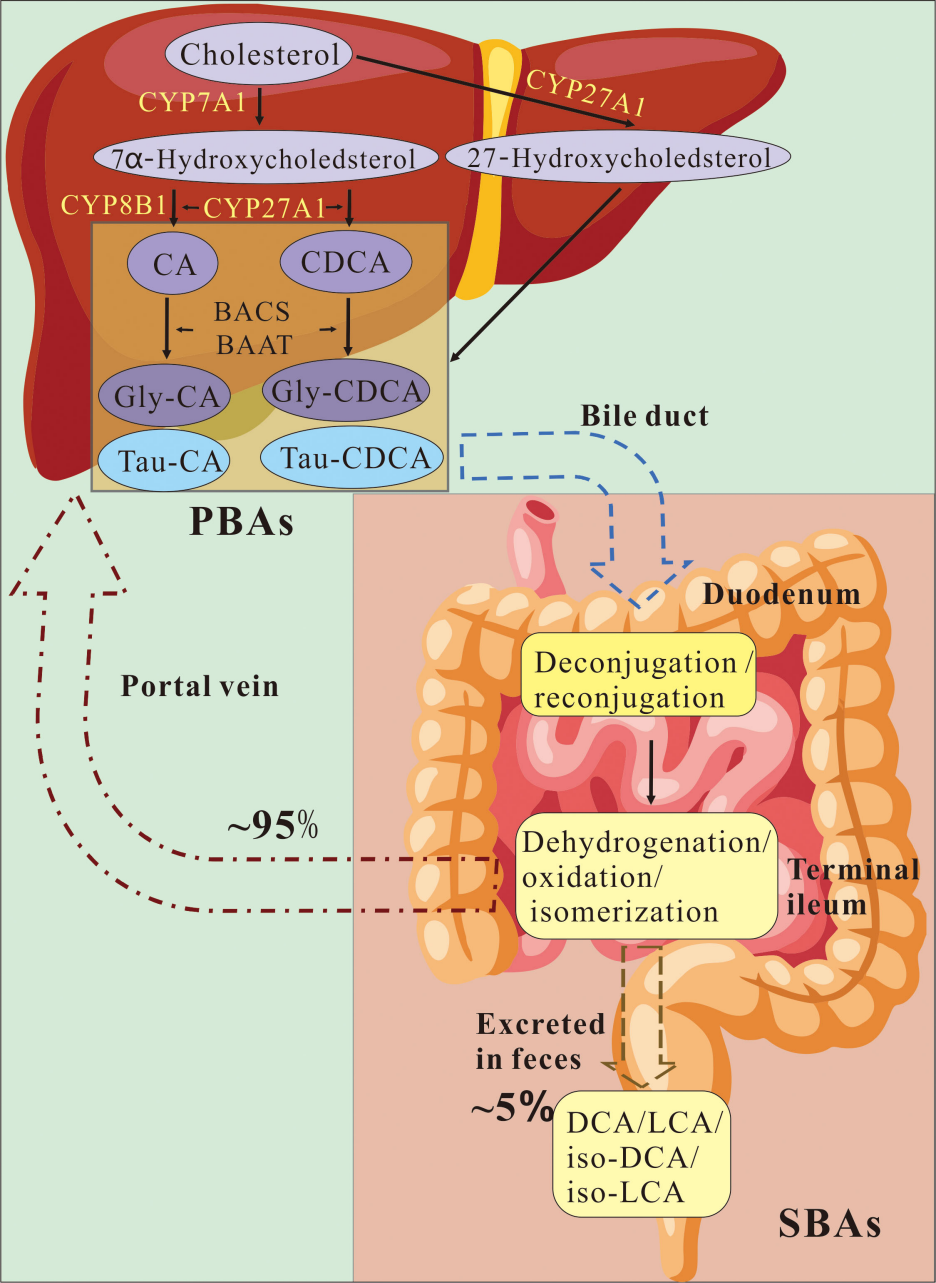
Illustration of the enterohepatic circulation process. In the liver, cholesterol is metabolized into primary bile acids (PBAs), cholic acid (CA) and chenodeoxycholic acid (CDCA), through two distinct pathways: the classical (or neutral) pathway and the alternative (or acidic) pathway. These PBAs are subsequently stored in the gallbladder and released into the duodenum via the bile duct following meals, where they undergo deconjugation and microbial-mediated reconjugation. Within the small intestine and colon, PBAs are converted into secondary bile acids (SBAs), deoxycholic acid (DCA) and lithocholic acid (LCA) along with their respective derivatives, through a series of microbial reactions that include dehydrogenation, oxidation, and isomerization. Approximately 95% of both PBAs and SBAs are reabsorbed in the terminal ileum through intestinal bile acid transporters. Following their exit from enterocytes, these bile acids are transported back to the liver via the portal vein, thereby completing the enterohepatic circulation and diminishing the need for *de novo* synthesis of PBAs. Only about 5% of bile acids are eliminated in the feces. CYP7A1, cytochrome P450 7A1; CYP27A1, cytochrome P450 27A1; CYP8B1, cytochrome P450 8B1; CA, Cholic Acid; CDCA, Chenodeoxycholic acid; BACS, Bile acid:CoA synthase; BAAT, bile acid-CoA: amino acid N-acyltransferase; BA, Bile acid; PBAs, primary bile acids; SBAs, secondary bile acids; LCA, Lithocholic acid; DCA, Deoxycholic Acid;.

Postprandially, bile acids released into the duodenum as mixed micelles comprising bile acids, cholesterol and phospholipids. These micelles facilitate the emulsification and absorption of dietary lipids (22). As the intestinal contents progress through the gastrointestinal tract, PBAs reach the ileum and colon, where 90%-95% of bile acids are reabsorbed in the terminal ileum and colon via the apical sodium-dependent bile acid transporter (ASBT)-mediated sodium-dependent active uptake (23). Then the ileal bile acid binding protein (IBABP) in the cytoplasm specifically binds to bile acids and mediates the intracellular transport of bile acids from the apical membrane to the basolateral membrane. Meanwhile, the heteromeric organic solute transporter α-β (OSTα-OSTβ) is responsible for the basolateral export of most bile acids, enter the portal circulation. Through two classes of transporters localized on the sinusoidal membrane, sodium taurocholate cotransporting polypeptide (NTCP; SLC10A1) and the organic anion transporting polypeptide (OATP), hepatocytes selectively take up the majority of conjugated and unconjugated bile acids in the portal vein, respectively, thereby maintaining the enterohepatic circulation (24) (**Figure 1**).

Approximately 3 grams of bile acids, which circulate through this enterohepatic cycle eight times daily, with only 0.2 to 0.6 grams requiring *de novo* synthesis by the liver to maintain the bile acid pool (25). Activation of the farnesoid X receptor (FXR) in the ileum and liver by recycled bile acids suppresses CYP7A1 expression, thereby limiting *de novo* PBAs synthesis and preserving cholesterol homeostasis (26). A small fraction (5-10%) of the bile acid pool escapes enterohepatic circulation; roughly 5% of PBAs reach the colon, and approximately 10% of the colonic bile acid pool is reabsorbed into the host (27). Recent evidence challenges the traditional notion that bile acid amination is restricted exclusively to the liver. Transcripts encoding bile acid-CoA:amino acid N-acyltransferase (BAAT), the enzyme catalyzing bile acid conjugation with amino acids, have been detected in various extrahepatic tissues-including the gallbladder, spleen, ovary, and brain, indicating a broader intrinsic capacity for bile acid amination (28). Furthermore, studies in germ-free mice have revealed significantly reduced levels of glycine-conjugated bile acids, underscoring the role of the gut microbiota in augmenting host bile acid conjugation (29). Complementary findings demonstrate that intestinal microbes can synthesize glycine-conjugated bile acids such as cholic acids and deoxycholic acids, which may function as biosurfactants to inhibit competing microorganisms (30). Collectively, these insights blur the conventional dichotomy between host-derived and microbe-derived bile acids, suggesting that, glycine-conjugated bile acids should not be exclusively classified as host-synthesized metabolites.

## 3 Transformation of SBAs

**Figure 2:**
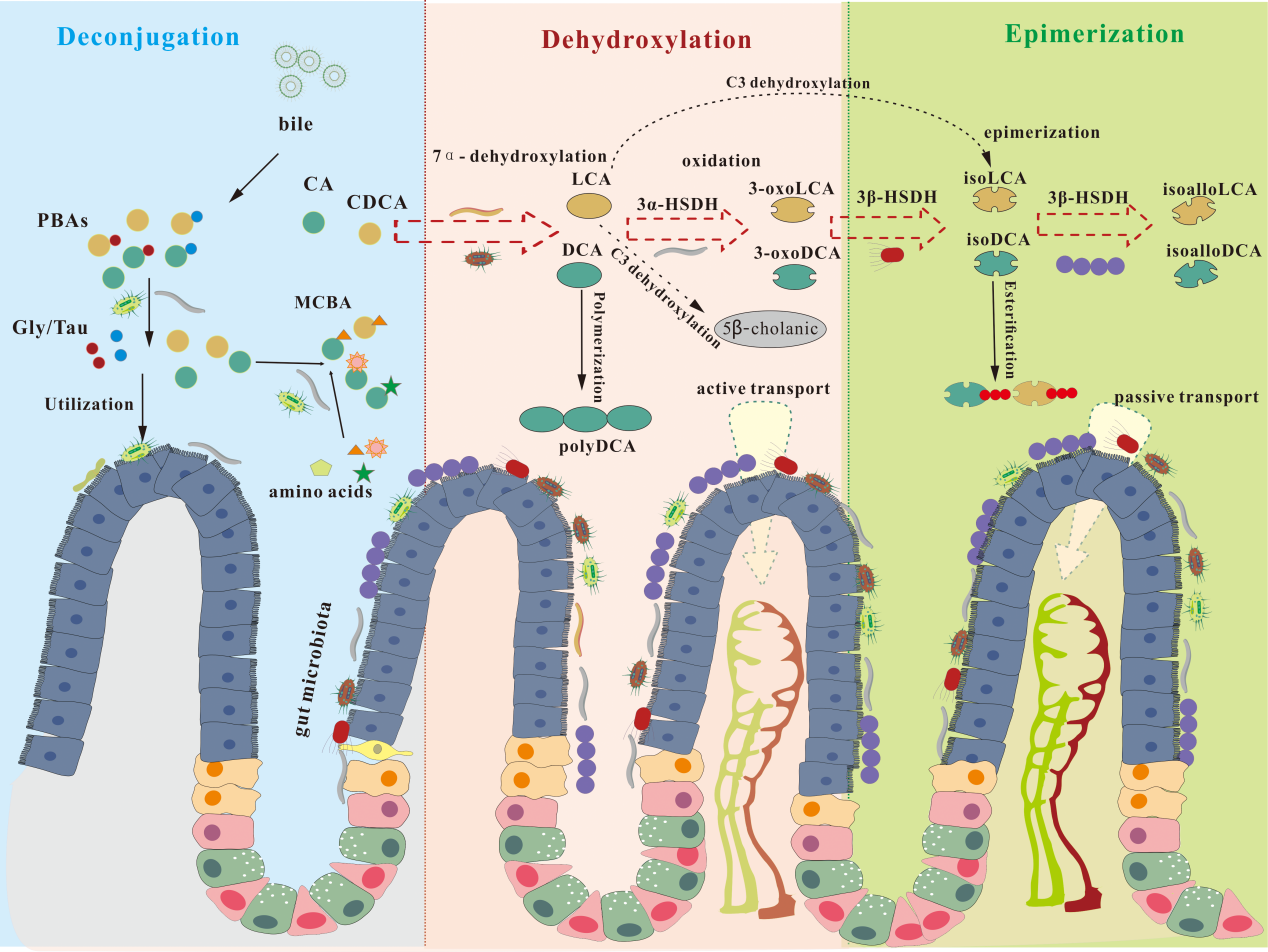
Bile acids biotransformation in the human gastrointestinal tract. Bile acids are secreted from the liver into the duodenum as mixed micelles, primarily in conjugated forms such as taurocholic acid (TCA) and glycocholic acid (GCA). In the intestine, gut microbiota initiate transformation via bile salt hydrolase (BSH)-mediated deconjugation, releasing free primary bile acids and enabling the uptake of glycine/taurine for microbial growth. Notably, BSH also catalyzes the reverse reaction, conjugating diverse amino acids to generate microbially conjugated bile acids (MCBAs). Free bile acids then undergo position-specific dehydroxylation. The major pathway is 7α-dehydroxylation, carried out predominantly by Bacillota and Clostridium cluster XIVa, converting CDCA to lithocholic acid (LCA) and CA to deoxycholic acid (DCA). Additional modifications include C3- and C12-dehydroxylation. Most bile acids are reabsorbed and returned to the liver. The remaining secondary bile acids (SBAs) undergo further microbial oxidation and epimerization.

The intestinal microbiota possesses a vast repertoire of functional genes that facilitate diverse biochemical modifications of SBAs (31). These gut microorganisms mediate the deconjugation and alteration of functional groups on PBAs while preserving the integrity of the core steroid ring structure. Among the various biotransformation pathways, 7α-dehydroxylation is recognized as a pivotal enzymatic process responsible for the generation of SBAs (31). Additional microbial modifications further enhance the structural diversity and functional complexity of the bile acid pool, thereby exerting significant effects on host physiology (40). The transformation of SBAs occurs within the intestinal environment through a series of microbiota-driven reactions, including deconjugation, dehydrogenation, oxidation, and isomerization of PBAs, collectively contributing to the intestinal SBAs pool (**Figure 2**). Beyond transformation mediated by the gut microbiota, the composition of the bile acid pool exhibits significant inter- and intra-individual variability, shaped by factors such as age, sex, and dietary interventions. As summarized in **Box 1**, these dynamic change underscore the profound influence of host physiology and gut microbiota on bile acid profiles and their potential roles in health and disease.

Box 1 **The impact of age and gender on the pattern of bile acid profiles.**

At the individual level, the bile acid pool is distinct and demonstrates variability at different time intervals throughout a single day (32). Conversely, the bile acid pool tends to be more uniform at the population level, with age and gender acting as significant factors for the systemic bile acid pool. PBAs are detected from infancy, including both those produced internally and those transferred through the umbilical cord (33). Tetrahydroxylated bile acids, which are the main type found in meconium, form a unique group that are often absent from the bile profiles of many adults (34). The exact timing of the shift from neonatal to microbially derived bile acids in the gut is not well understood. In older adults, the bile acid profile is mainly dominated by conjugated bile acids (35). Studies on centenarians have revealed an increase in oxidation-reduction reactions and enantiomeric changes of bile acids driven by the gut microbiota, leading to higher levels of iso-LCA, 3-oxo-LCA, allo-LCA, 3-oxo-allo-LCA, and iso-allo-LCA (36). These bile acids are highly hydrophobic and can activate cellular receptors at nanomolar concentrations, influencing mitochondrial function and modifying cellular energy balance, which may help slow down the aging process. For instance, the buildup of bile acids in yeast mitochondria has been linked to an anti-aging cellular state (37). Research across different species further supports the beneficial effects of serum LCA on lifespan. Caloric restriction diets encourage the growth of LCA producer in gut, leading to higher serum LCA levels. LCA in serum enhanced muscle function through activation of the AMP-activated protein kinase (AMPK) pathway, thereby extending health span in *Caenorhabditis elegans, Drosophila melanogaster, and Mus musculus* (1). This phenomenon may explain one of the unique characteristics of SBAs found in populations with exceptional longevity.

Sex differences are another key factor affecting systemic bile acid profiles. Studies show that from three weeks of age, female mice have higher levels of conjugated bile acids and SBAs in serum, possibly due to differences in liver bile acid synthesis, as male mice have elevated conjugated bile acids in liver (38). A significant sex-related difference in systemic bile acid levels is also observed in pregnant mice. Estrogens regulating bile acid balance during pregnancy by increasing levels of 17β-estradiol metabolites and suppressing the activity of the small heterodimer partner (SHP), leading to an abnormal rise in bile acid levels (39).

Historically, SBAs were primarily characterized by the microbial removal of the 7α-hydroxyl group from PBAs (41). However, advancements in analytical techniques such as liquid chromatography-mass spectrometry (LC-MS) and omics, approaches have led to the identification of numerous additional microbial pathways involved in SBAs transformation (42). It is now well established that SBAs represent a class of metabolites extensively modified by enzymes derived from gut microbes (43). Unsulfated Lithocholic acid (LCA) and Deoxycholic acid (DCA) participate in the enterohepatic circulation similar to PBAs: in the colon, these SBAs are passively absorbed in the colon and enter the portal venous system (44). Within the liver, they undergo extensive metabolic processing, including reconjugation, rehydroxylation, epimerization, and sulfation, before being secreted into bile and re-entering the enterohepatic cycle after transformations (45). The fraction of SBAs not absorbed in the colon is ultimately excreted via feces. Under normal physiological conditions, plasma concentrations of SBAs remain very low (<10 μM), primarily due to efficient hepatic extraction (46). Notably, humans exhibit substrate-specific metabolic preferences: LCA is more readily sulfated, enhancing its hydrophilicity and facilitating excretion, whereas a portion of DCA undergoes preferential rehydroxylation, producing more hydrophilic derivatives that contribute to the maintenance of enterohepatic homeostasis (47).

### 3.1 Deconjugation

The initial documentation of the deconjugation of PBAs by a consortium of fecal bacteria and specific microbial isolates dates back to 1936 (48). Bile Salt Hydrolase (BSH, E.C.3.5.1.24), an enzyme belonging to the bile acid glycine hydrolase family, has been extensively studied as a key mediator of microbial bile acid metabolism. Numerous comprehensive reviews addressing this subject are available in the literature (49, 50). The *bsh* gene has been identified in a wide range of gut microorganisms, particularly among Gram-positive genera such as *Clostridium*, *Lactobacillus*, *Bifidobacterium*, and *Enterococcus* (49). In contrast, *bsh* occurrence is relatively rare among Gram-negative bacteria, with current evidence suggesting its presence in specific species within the genus *Bacteroides* (51). Additionally, *bsh* genes have been detected in gut-associated archaeal species (52). Phylogenetic analyses suggest that horizontal gene transfer may have facilitated the acquisition of the *bsh* gene from *Bacillus* species to intestinal methanogenic archaea in the human gut (53).

The BSH-catalyzed deconjugation of PBAs is hypothesized to promote the proliferation of gut microbial communities by liberating amino acids that serve as sources of carbon, nitrogen, and sulfur, thereby conferring a growth advantage to the microorganisms involved (54). Moreover, this deconjugation process is proposed to enhance bacterial tolerance to bile acids, potentially facilitating colonization and persistence within the intestinal milieu (55). Certain microorganisms, such as *Lactobacillus* spp., incorporate bile acids into their cell membranes, which improves membrane tensile strength and fluidity, thereby aiding in evasion of immune detection and clearance (56). In healthy vertebrates, deconjugation of PBAs is a substrate-limited process and primarily complete in the gut (57). Compared to their conjugated counterparts, free bile acids exhibit lower polarity and reduced absorption efficiency, resulting in increased fecal excretion (58). Consequently, elevated concentrations of free bile acids stimulate *de novo* bile acid synthesis from cholesterol or enhance reverse cholesterol transport in the liver, mechanisms that contribute to the reduction of serum cholesterol levels (59).

### 3.2 7α-dehydroxylation

The deconjugation of PBAs is a prerequisite for 7α-dehydroxylation, whereas inhibition of deconjugation activity leads to an accumulation of fecal PBAs and a concomitant reduction in SBAs in murine models (60). The *bai* operon within the intestinal microbiota mediates the 7-dehydroxylation of PBAs. This operon comprises eight genes- *BaiA2*, *BaiB*, *BaiCD*, *BaiE*, *BaiF*, *BaiN*, *BaiH*, and *BaiO* —each corresponding to distinct enzymatic steps involved in the conversion of PBAs to SBAs (61). Members of the phylum Bacillota, including families such as *Ruminococcaceae*, *Peptostreptococcaceae*, *Lachnospiraceae*, and *Oscillospiraceae*, exhibit the 7-dehydroxylation activity toward PBAs in the human gut. These taxa, however, constitute relatively low-abundance populations within the gut microbiota, with densities ranging from 10^3 to 10^7 colony-forming units per gram of wet fecal matter (43, 50, 62). Investigations of complex microbial consortia, comprising from a few to approximately one hundred species, have demonstrated that despite their low abundance, these populations are critical for the biosynthesis of DCA and LCA (63–66). Within this context, members of *Clostridium* cluster XIVa have been extensively studied with respect to the *bai* operon. Cellular extracts of *Clostridium scindens* VPI 12708 have revealed numerous bile acid intermediates along the multi-step, branched pathway converting CA to DCA, including the isomer alloDCA. Furthermore, reverse genetic approaches have enabled the successful cloning of the *bai* operon (~12 kilobases) from this strain (67).

The 7α-dehydroxylation of CDCA and CA produces LCA and DCA, respectively. Additionally, CDCA can be converted into UDCA, in humans (68). Reflecting interspecies differences in the composition of the PBAs pool, the rodent gut microbiota 7α-dehydroxylate α/β-muricholic acids (MCAs) to generate ωMCA, hyodeoxycholic acid (HDCA), and murideoxycholic acid (MDCA) (69). In contrast, although the human gut microbiota can colonize germ-free mice, it lacks the capacity to metabolize β-MCA (63, 70). This specificity implies a co- evolutionary relationship between the bai -encoded enzymes and the endogenous PBAs of host. Moreover, unlike humans, rodents express hepatic cytochrome P450 enzyme CYP2A12, which facilitates 7α-rehydroxylation of SBAs back to PBAs, thereby resulting in a higher PBAs to SBAs ratio (71).

The gut microbiota must delicately manage reducing equivalents to thrive within the highly reduced, anaerobic environment of the gastrointestinal tract (72). The dehydroxylation of PBAs serves a dual purpose: it partially fulfills the requirements of microorganisms for regulating reducing equivalents and simultaneously provides a survival advantage to SBAs producers by increasing the concentration of toxic metabolites that suppress competing microbial populations while enhancing mutualistic interactions (8, 73). The 7-dehydroxylation of PBAs is characterized as a redox reaction in which PBAs act as electron acceptors, culminating in a total net reduction of two electrons (74). Allo-SBAs are generated during the microbial biosynthesis of DCA and LCA, a process the *Bai* operon involved (41). The enzyme BaiE catalyzes the formation of intermediates 3-oxo-4-DCA or 3-oxo-4-LCA following the 7α-dehydration, which are subsequently reduced by *BaiP* or *BaiJ* (bile acid 5α-reductase) in conjunction with *BaiA* to synthesize allo-DCA and allo-LCA (75). Additionally, alternative pathways involving microbial 3α-hydroxysteroid dehydrogenases (HSDHs), 5β-reductase, and 5α-reductase may also contribute to allo-bile acids formation through metabolic equilibrium (36). However, the exact contributions of these pathways to the intestinal bile acid profile and the considerable intra- and inter-individual variability in allo-bile acid concentrations remain to be fully elucidated.

### 3.3 C3- and C12- dehydroxylation

In addition to the extensively studied C7-dehydroxylation, the human gut microbiota also facilitates dehydroxylation at the C3 and C12 of PBAs. Nevertheless, a thorough mechanistic elucidation of these pathways and identification of the microbial taxa involved remain limited, primarily due to methodological challenges in bile acid tracing (76). *In vitro* investigations have demonstrated that human fecal microbiota can convert sulfated lithocholic acid (LCA-3S) into isolithocholic acid (isoLCA), Δ³-cholenic acid, and ultimately 5β-cholanic acid (77). The antibiotic vancomycin inhibits the metabolism of 3-sulfate-LCA, whereas interventions that promote the germination of Gram-positive spores enhance this metabolic process (77). Studies also shown that Clostridia isolates independently catalyze this conversion in pure culture (77). The cleavage and inversion of the carbon-oxygen bond, a reaction characteristic of arylsulfatase enzymes, is hypothesized to facilitate isoLCA formation, potentially representing an alternative biosynthetic route (77). Subsequently, isoLCA undergoes dehydroxylation to form Δ^3^-cholenic acid, which is further reduced to 5β-cholanic acid. It is plausible that the trans-elimination of the sulfate ester leads to Δ^3^-cholenic acid formation, followed by its reduction to 5β-cholanic acid. Notably, Garcia et al. (2022) reported the direct conversion of CDCA to 7α-hydroxy-5β-cholan-24-oic acid in human fecal suspensions, suggesting C3-dehydroxylation may precede-rather than follow-LCA formation during CDCA metabolism (78).

The process of C12-dehydroxylation further complicates the metabolic pathways of DCA, LCA, and their derivatives. Isolates of *Bacteroides* have been shown to convert CA to CDCA via C12-dehydroxylation; however, this capability diminishes with extended *in vitro* cultivation (79). Isotopic tracer studies provide *in vivo* evidence supporting microbial-mediated (de)hydrogenation at the C11-C12 bond, as indicated by the instability of [11,12-³H] labels in CDCA and LCA during human enterohepatic circulation indicates microbial-mediated (de)hydrogenation at the C11-C12 bond (80). While the pathways underlying C3-dehydroxylation have been partially elucidated, the mechanisms governing C12-dehydroxylation remain poorly understood. Therefore, comprehensive investigations employing advanced analytical techniques are imperative to clarify the enzymatic processes involved, identify the responsible microbial taxa, and quantify the role of C12-dehydroxylation in maintaining bile acid homeostasis.

### 3.4 Microbial conjugation

Host-derived conjugated bile acids are conventionally characterized by taurine or glycine side chains; however, recent investigations have revealed the presence of non-canonical bile acid conjugates in humans and specific pathogen-free (SPF) mice. These microbially conjugated bile acids (MCBAs) incorporate amino acids such as isoleucine, leucine, phenylalanine, and tyrosine, and are found in both PBAs and SBAs (81). Their absence in germ-free mice substantiates the role of microbial activity in their biosynthesis (30, 82). Recent twin studies have elucidated that *bsh* are instrumental in the intestinal synthesis of MCBAs via a reversible hydrolysis-coupling mechanism (83, 84). While taurine serves as a cofactor in microbial bile acid amidotransferase activity, facilitating amidation with diverse amines, the precise mechanism of bile acid activation requires further elucidation (30, 85). Reverse metabolomics has significantly expanded the known repertoire of microbially conjugated bile acids (MCBAs) to include conjugates with citrulline, glutamate, histidine, lysine, and threonine, and even polyamines like putrescine, cadaverine, and spermidine, which form C-24 amide bonds (42).

The levels of MCBAs exhibit pronounced dietary dependence and distinct spatial patterning along the gastrointestinal tract. Specifically, MCBA concentrations are markedly elevated in individuals consuming meat-rich diets (42). Spatial quantification in healthy mice reveals a steep gradient, with levels of compounds such as cholic acid-phenylalanine amide peaking in the jejunum (248 mg/kg) and declining sharply in distal segments-ileum (46 mg/kg), cecum (3 mg/kg), and colon (6 mg/kg) (82). This compartmentalization underscores their predominant retention within the intestinal lumen rather than fecal excretion. Furthermore, MCBA levels correlate positively with concentrations of free bile acids (86). The expansion of the bile acid pool by MCBAs is driven by the diverse repertoire of BSH enzymes encoded by dominant gut bacterial phyla, which generate thousands of structurally unique cholic acid derivatives (87).

Functionally, MCBAs exhibit distinct physiological activities that differ from their bile acid cores, suggesting a potential role in mediating host-microbiota communication (10, 88). CDCA has been identified as the most potent endogenous ligand for the FXR, and conjugation with phenylalanine or tyrosine enhances FXR activation by twofold and sixty-ninefold, respectively (82). Amino acid side chains are involved in modulating the recognition and binding interactions between bile acids and their receptors, and microbiota-driven alterations in these side chains may influence the agonistic or antagonistic activities of bile acids on their receptors (88). These findings support the concept that the gut microbiota shapes the bile acid pool, which is subsequently interpreted by microbial communities, bile acid receptors, and transporters, thereby influencing the physiological functions of both the microbiota and the host (89, 90). The fundamental structure of MCBAs-characterized by the curved A/B ring of cholic acid and the amphipathic nature of the bile acid nucleus—facilitates access to intracellular host sites and organelles, while the associated side chains provide detachable chemical information (10). How alterations in the gut bile acids translate into systemic immune modulation via MCBA is an area of active inquiry (91). The application of advanced enrichment methodologies, such as click chemistry, combined with artificial intelligence-based predictions of novel bile acid receptors and their signaling pathways, holds promise for elucidating the role of MCBAs as microbial-derived chemical mediators within the host (92, 93).

### 3.5 Oxidation and epimerization

Recent advancements in LC/MS methodologies have elucidated a variety of microbial metabolites originating from SBAs modifications, including hydroxyl oxidation and epimerization at the C3, C7, and C12 positions. Notable metabolites identified include oxo-deoxycholic acid (oxoDCA), isoLCA, isodeoxycholic acid (isoDCA), and isoallolithocholic acid (isoalloLCA) (94, 95). The generation of oxo-cholic acid intermediates is a critical step in hydroxy epimerization, facilitating the interconversion of bile acids between α- and β- configurations. This process requires the coordinated activity of two distinct HSDHs that differ in both their positional specificity and stereochemical properties, and is mediated by microorganisms from diverse taxa. For instance, *Clostridium absonum* is capable of epimerizing bile acids independently due to the presence of both 7α- and 7β-HSDHs (96). Moreover, co-cultivation of two intestinal bacteria expressing either 7α-HSDH or 7β-HSDH can achieve similar epimerization (97, 98). A recent investigation has also identified novel 3-O-acylated cholic acids produced via 3α-hydroxy acylation catalyzed by *Christensenella minuta*, utilizing short-chain fatty acids (SCFAs) serving as acyl donors (99).

Several microbial species, *Eggerthella lenta*, *Blautia producta*, *Clostridium absonum*, *Clostridium perfringens*, *Clostridium paraputrificum*, *Escherichia coli*, *Bacteroides fragilis*, and *Ruminococcus gnavus*, have been recognized as carriers of enzymes that mediate the oxidation and reduction of bile acids (100–102). Correspondingly, the *hsd* genes responsible for encoding these enzymes have been identified and characterized across various strains within these species. Current research efforts are directed towards isolating additional bacterial strains capable of bile acid oxidation and reduction (30, 103).

The oxidation and reduction of hydroxyl groups on bile acids constitute a microbial mechanism that modulates the redox potential within the intestinal environment. *In vitro* studies of intestinal microbiota demonstrate that elevated oxygen levels in the culture lead to the accumulation of oxo-bile acids (104, 105). Although the colonic environment in the host is generally reducing, the mucosal surface exhibits a relatively higher redox potential, which may facilitate oxidation-reduction reactions resulting in oxo-bile acid formation. Conversely, within the colonic lumen, where the redox potential is lower (approximately -200 to -300 mV), these reactions may preferentially drive the reduction of oxo-bile acids (106).

### 3.6 Fatty acid esterification, sulfation, and polymerization

A growing body of research has identified a substantial number of bacterial modifications to bile acids, with at least 5,576 distinct alterations documented. Fatty acid esterification, sulfation, and polymerization of bile acids are considered to reduce the toxicity of bile acids (42). Bile acid esters, which primarily arise from modifications involving alcohols, SCFAs, and long-chain fatty acids in fecal samples from healthy individuals, accounting for approximately 10-30% of the total bile acids, predominantly in the form of isoDCA and isoLCA (107). Nevertheless, the quantification of these esterified forms in fecal samples is infrequently conducted in contemporary studies. The methylation of DCA appears to result from the transfer of a methyl or methoxy group from C1 to C24 (85, 92). The bile acids esterification facilitated by *Lactobacillus*, *Eubacterium*, and *Bacteroides*, depend on the ethanol addition, indicating the involvement of cross-feeding among microbial communities in the modification of bile acids (92, 108, 109).

The intestinal microbiota mediates the esterification of bile acids, primarily generating fatty acid esters with isoDCA and isoLCA at the C3 position, utilizing both long-chain (C16, C18) and SCFAs (107). Beyond simple esterification, DCA can undergo oligomerization via its C24 carboxyl and 3α-hydroxyl groups, forming polyester chains (110). These modifications are broadly considered microbial detoxification strategies that precipitate bile acids and fatty acids, thereby reducing their bioavailability. A distinct form of esterification involves dicarboxylic acids. For instance, *Bacteroides uniformis* produces 3-succinylated bile acids via the enzyme BAS-suc. Rather than acting directly on bile acid receptors, these succinylated derivatives promote the proliferation of *Akkermansia muciniphila*, which in turn activates toll-like receptor 4 (TLR4), contributing to the mitigation of liver injury (92).

Bile acid sulfonation serves as a critical host detoxification mechanism for SBAs (111). Notably, dehydroxylated derivatives such as DCA and LCA, which may constitute up to 25% of the biliary bile acid pool, cannot be reconverted to PBAs in humans, a metabolic limitation that underscores the importance of sulfonation for their elimination (P. B. Hylemon et al., unpublished data) (68). In the human liver, LCA undergoes sulfation at the 3-hydroxyl position and conjugation at the C-24 prior to its resecretion into bile. Due to the limited absorption of bile acid sulfates by the host, 3-sulfate-LCA is predominantly excreted in feces and typically does not accumulate within the enterohepatic circulation (112).

The SBAs sulfonation attributed to the host enzyme sulfotransferase family 2A member 1 (SULT2A1), which catalyzes the formation of 3-sulfo-lithocholic acid (3-sulfo-LCA) to mitigate the cytotoxic effects of LCA on liver (111). Recent study has identified a novel host-conjugated bile acid-methylcysteamine (BA-MCY) that is prevalent in the intestine. Vanin 1 (VNN1), a pantetheinase in the intestinal tract, facilitates the generation of BA-MCY, and mediated the bile acid signaling (93). The intestinal absorption of LCA has been shown to induce the sulfonation of other bile acids in the liver, such as cholic acid-7-sulfate, in both human and murine models (113). Previous work has revealed that sulfotransferases (BtSULT, BT0416) produced by *Bacteroides* commensal bacteria possess the ability to selectively modify bile acids, indicating a potential microbial contribution to the formation of sulfated bile acids within the host (114). Additionally, intestinal microorganisms can desulfate bile acids via aryl sulfatase enzymes (115, 116). Although the specific microbial sulfatases responsible for this activity have yet to be identified, it is posited that the desulfurization processes of the gut microbiota are linked to genera *Peptococcus*, *Clostridium*, *Pseudomonas*, and *Fusobacterium* (117, 118). Leveraging protein language models (ESM-2) and deep learning architectures, Ding et al. (40) developed Bile acid Enzyme Annotation Tool (BEAUT) to systematically identify BAs metabolic enzymes within gut metagenomic datasets. This framework facilitated the prediction of 600,000 BA-metabolizing enzymes, revealing extensive enzymatic potential for bile acids transformations in the gut microbiome. Applying this approach, the authors identified a 3-acetylated deoxycholic acid synthase (ADS) encoded by *Bacteroides ovatus* as responsible for synthesizing 3-acetyl-deoxycholic acid (3-acetoDCA), a previously uncharacterized SBA. This finding underscores the vast, uncharted landscape of enzymatic strategies governing SBAs modifications. Consequently, the continued development of AI-driven platforms, exemplified by BEAUT, is poised to revolutionize the elucidation of these intricate metabolic networks (40).

## 4 Receptors of bile acid

**Figure 3:**
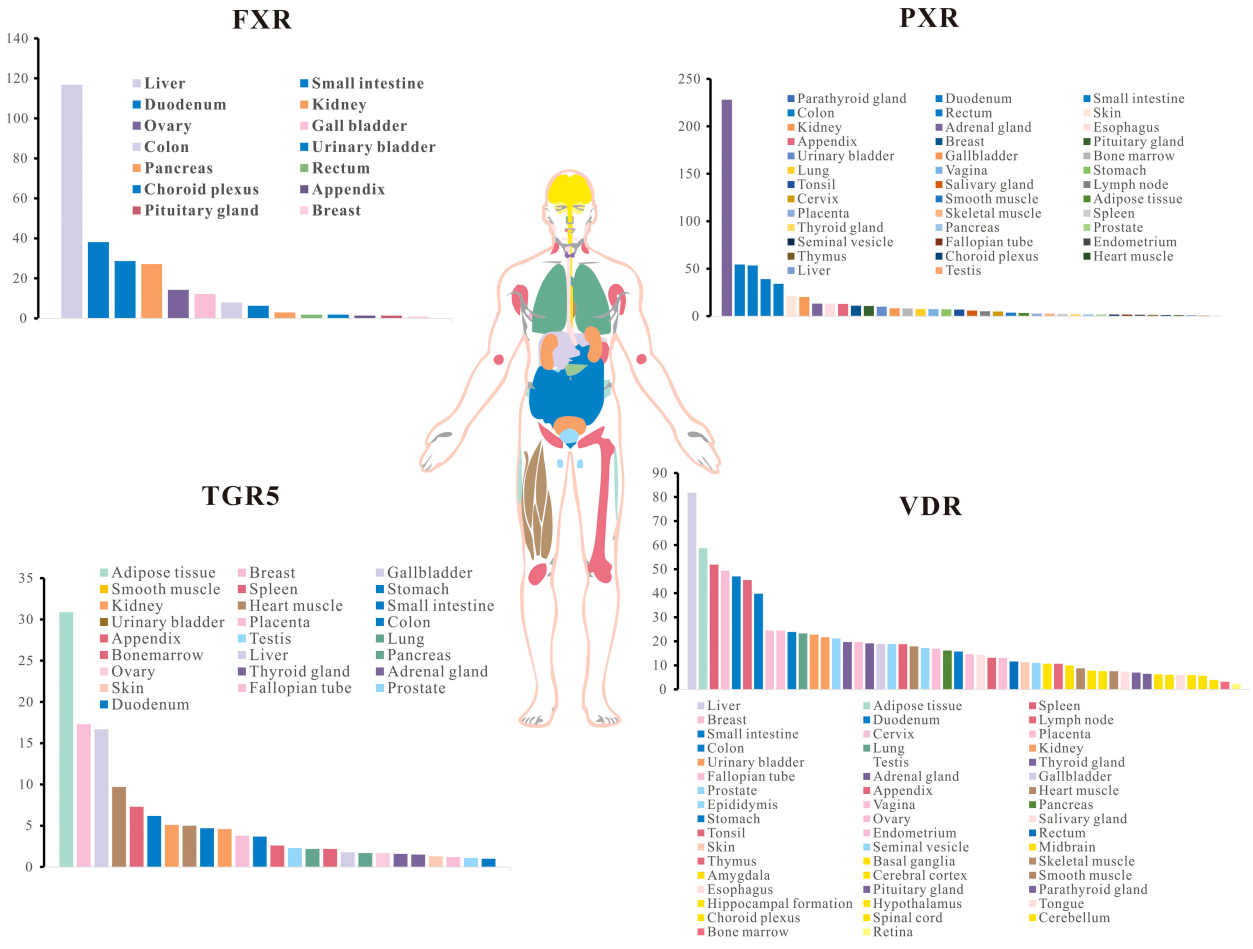
Gene expression of classical bile acid receptors in human tissues. (from “The Human Protein Atlas” (https://www.proteinatlas.org/).

Bile acid receptors distributed across various tissues (**Figure 3**) and respond to both PBAs and SBAs, playing critical roles in the regulation of bile acid synthesis, gastrointestinal motility, and immune system (**Table 1**) functions (151–154). These receptors are broadly classified into nuclear receptors and cell membrane receptors, each capable of recognizing bile acid ligands (14). The nuclear receptors identified include the vitamin D receptor (VDR), pregnane X receptor (PXR), constitutive androstane receptor (CAR), and farnesoid X receptor (FXR). Cell membrane receptors primarily consist of G protein-coupled receptors such as G Protein-coupled Bile Acid Receptor 1 (GPBAR1, TGR5), and sphingosine-1-phosphate receptor 2 (S1PR2) (155, 156).

**Table 1:** Distribution and function of classical bile acid receptors in immune cells.

Bile acids receptor	Natural ligands	Immune cell distribution	Function
FXR (NR1H4)	Agonists: CDCA > CA > LCA > DCA (119) Antagonists: UDCA (120); 7-keto-LCA (121); α/βMuricholic acid (122) Agonists: Phe/Tyr/Trp/Glu-CAs Antagonists: Leu/Ser/Ala-CAs (88)/ BA–methylcysteamine (93)	Monocytes/Macrophages	FXR negatively regulates the NLRP3 inflammasome through physical interactions with NLRP3 and caspase-1, consequently reducing the levels of IL-1β (123). Activation of FXR leads to a decrease in the levels of inflammatory cytokines, simultaneously suppresses the activation of NF-κB, and promotes the polarization of M2 macrophages (124).
		Dendritic cells (DCs)	The activation of FXR in splenic DCs promotes their retention in the spleen (125).
		innate lymphoid cells (ILCs)	Activation of the FXR in ILCs leads to the upregulation of the nuclear receptor REVERBα, inhibit the expression of IL-17; Additionally, FXR facilitates the accumulation of ILC precursors while simultaneously inhibiting their differentiation into mature ILC3s (126).
		natural killer (NK)T cells	The activation of FXR in NKT cells promotes the upregulation of the short heterodimer partner (SHP), which in turn leads to the suppression of osteopontin expression (127).
		CD8^+^ T	Effector T cells detect glucose levels through the FXR, allowing for a dynamic modulation of T cell populations in response to nutritional availability (128).
TGR5 (GPBAR1)	LCA > DCA > CDCA > UDCA > CA Antagonists: Leu/Phe/Tyr/ Glu/Trp/Ala/Ser-CA (88)	DCs	SBAs induce the expression of FXR in DCs and, via the cAMP/PKA signaling pathway, suppress the expression of NF-κB and its associated downstream cytokines (129).
		NKT cells	TGR5 maintains the anti-inflammatory phenotype of liver NKT cells by upregulating the expression of IL-10 through CREB (cAMP response element-binding protein) phosphorylation (130).
		CD8^+^ T	TGR5 facilitates the activation of CD8^+^ T cells through the mTOR/oxidative phosphorylation signaling pathway (131).
		Macrophages	TGR5 facilitates the M2 polarization of tumor-associated macrophages (TAMs) through the cAMP-STAT3/STAT6 signaling pathway, which in turn contributes to the inhibition of CD8^+^ T cell activity (132). Sauchinone activates TGR5 on macrophages, subsequently inhibits M1 polarization and reduces the expression of TNF-α, IL-6, and iNOS via the cAMP/PKA signaling pathway (133).
VDR (NR1H1)	Agonists: LCA and derivatives (134)	Monocytes/ Macrophages	Research has demonstrated that the VDR is situated on the cell membrane of macrophages, playing a crucial role in the development of LPS tolerance in these immune cells (135). Within the tumor microenvironment, the activation of the VDR facilitates the polarization of macrophages towards the M1 phenotype, concurrently suppressing the M2-type macrophages (136).
		DCs	Activation of the VDR leads to the induction of tolerogenic properties in myeloid DCs, facilitated by the upregulation of the inhibitory receptor ILT3, the enhancement of CCL22 secretion, and the inhibition of NF-κB nuclear translocation (137).
		NKT cells	The presence of the VDR on hematopoietic cells is essential for the maturation of T-bet in invariant NK T (iNKT) cells (138).
		CD4^+^ T cells	When vitamin D is present, the expression of the VDR in CD46^+^ T cells modulate epigenetic modifications of Th1 cells that enable the transformation of pro-inflammatory IFN-γ^+^ Th1 cells into anti-inflammatory IL-10^+^ cells (139).
		CD8^+^ T cells	Vitamin D signaling plays a crucial role in shaping the overall trajectory of anti-infective immunity by regulating various aspects of cytotoxic T lymphocyte (CTL) differentiation, including granzyme B expression, survival mechanisms such as Bcl-2 expression, the diversity of epitope recognition, and the localization of these cells within tissues (140).
PXR (NR1H2)	Agonists: 3k-LCA; LCA; CDCA; DCA; CA (141)	Monocytes/ Macrophages	The activation of PXR suppresses the M1 polarization of macrophages while facilitating M2 polarization through the activation of STAT6 (142).
		B cells	3-indoleacetic acid (IAA) and LPS stimulate B cell activation through the PXR and toll-like receptor 4 (TLR4), facilitated the nuclear translocation of the PXR/NF-κB/RXR complex, which subsequently enhances the expression of IL-35 in B cells (143).
LXRα/β	Agonists: 6α-hydroxylated bile acids (144)	Monocytes/ Macrophages	The suppression of LXR results in an increased expression of the musculoaponeurotic fibrosarcoma oncogene homolog B (MAFB) within macrophages facilitates the development of anti-inflammatory gene expression and functional characteristics during GM-CSF-dependent differentiation of macrophages originating from pathological conditions, as well as in macrophages derived from human monocytes (145).
		DCs	Tumors impede the migration of maturing DCs to lymphoid organs through the activation of LXR, which results in the downregulation of CCR7 expression on these cells (146). The activation of LXR results in the suppression of DC maturation and migratory capacity induced by TLRs (147).
		NKT cells	The presence of LXRα in iNKT cells increases the susceptibility of mice to concanavalin A (148).
		Th cells	LXR mediates the T cell receptor (TCR) and Wnt/β-catenin signaling pathways, thereby inhibiting the transcription factor TCF-1, impedes the differentiation of Tfh cells (149). LXR inhibits IL-17 transcription and hinders Th17 cell differentiation by binding to the E-box in the IL-17 promoter via sterol regulatory element-binding protein 1 (SREBP-1), and interacting with the transcriptional activator Ahr (150).

Among nuclear receptors, FXR is recognized as the master bile acid sensor in humans, with well-characterized roles in maintaining enterohepatic homeostasis (154). Host-derived PBAs typically act as FXR agonists, whereas many SBAs, such as isoDCA, DCA, and LCA, function as antagonists, inhibiting CDCA-induced FXR transcriptional activity by 30–50% in immune cells (157). FXR activation in intestinal epithelial cells induces fibroblast growth factor 19 (FGF19), which, upon reaching the liver, represses the bile acid biosynthesis enzymes CYP7A1 and CYP8B1. Concurrently, hepatic FXR upregulates the small heterodimer partner (SHP), further modulating bile acid production and export (158). Beyond metabolic regulation, FXR exerts anti-inflammatory effects by inhibiting nuclear factor-κB (NF-κB) nuclear translocation, thereby dampening proinflammatory cytokine production (159). Additionally, BA-MCY suppresses FXR activity via a thiol-dependent mechanism, suggesting a synergistic interaction between host and microbial bile acids within the FXR signaling pathway (93).

The VDR, evolutionarily conserved across mammals (160), is activated by LCA and its derivative 3-oxo-LCA (ligands of PXR) are implicated in hepatoprotection (141, 161). The binding of LCA to the VDR involves a dual-ligand configuration: LCA interacts with conserved amino acid residues, specifically zTyr175 and zSer306, within the ligand-binding pocket (LBP), inducing a conformational change. This structural alteration facilitates the formation of direct hydrogen bonds between surface-active sites, zSer263 and zGln267, located in the H2-H3 loop region, and the hydroxyl group on the A ring of LCA, culminating in full activation of the VDR (162). Functionally, VDR signaling enhances intestinal barrier integrity by upregulating tight junction proteins and suppresses the NF-κB pathway, collectively mitigating intestinal inflammation (134). In murine models, CAR expression in ileal T cells promotes their survival in bile acid-rich environments (163).

TGR5 is widely expressed across multiple organs and, in contrast to FXR, exhibits a stronger responsiveness to SBAs compared to PBAs (164, 165). It features a hydrophobic binding pocket approximately 15 Å in diameter, which enables the anchoring of bile acids through a combination of hydrophobic and polar interactions. Within the tripartite leucine cluster comprising residues L244^6·55^, L263^7·36^, and L266^7·39^), the residue L263^7·36^ is positioned at the base of the orthosteric binding site. Its side chain contributes significantly to the formation of a hydrophobic cavity that directly interacts with the amino acid-conjugated moiety at the C24 position of bile acids, serving as a key structural determinant for the recognition of these conjugated groups. Taurine-conjugated bile acids demonstrate a low EC_50_ (100 nM) in activating TGR5, whereas free SBAs preferentially stimulate the TRG5/β-arrestin signaling pathway, attributed to the reduced interaction of free SBAs with L263^7·36^, which leads to the exposure of extracellular loop 3 (ECL3) and thereby promotes β-arrestin recruitment (166). The activation of TGR5 also elevates intracellular cyclic adenosine monophosphate (cAMP) levels, which subsequently activates protein kinase A (PKA)-mediated signal transduction and modulates energy metabolism (167). Another key membrane receptor, S1PR2, is predominantly hepatic and activated by conjugated bile acids. Upon activation by conjugated bile acids, S1PR2 triggers the epidermal growth factor receptor (EGFR)/insulin receptor (IR) pathway, enhances sphingosine-1-phosphate (S1P) production, and inhibits histone deacetylases (HDACs). This cascade subsequently regulates the expression of FXR, thereby promoting bile acid synthesis (155).

Beyond the well-established canonical bile acid receptors, several novel receptors have recently been identified that interact with specific bile acids. Notably, 3-oxo-LCA and its dehydrogenated derivatives (3-oxo-Δ5-LCA, 3-oxo-Δ4-LCA, 3-oxo-Δ4,6-LCA) have been characterized as potent antagonists of the human androgen receptor (hAR), exhibiting IC_50_ values ranging from 99.7 to 443 nM (168). The gut microbial HSDHs and other dehydrogenases precisely modulate the androgen receptor antagonistic activity of these bile acids by altering the position of double bonds, promoting planarization of the A/B rings, and maintaining the presence of a C3-ketone group (168). Emerging evidence points to a potential role of Mas-related G protein-coupled receptor family member E (MRGPRE) serving the specific receptor for tryptophan-conjugated cholic acid (169). The hydroxyl groups at the C7 and C12 positions are critical for recognition via the transmembrane domains TM3 and TM7 of MRGPRE, underscoring the significant role of microbial bile acid modifications in receptor recognition (169). Additionally, the plasma membrane calcium ATPase (PMCA4) present in CD8^+^ T cells has been identified as the receptor for DCA, in colon cancer murine models, although the precise binding site remains to be elucidated (170). LCA has also been shown to bind to TULP3 (TUB-like protein 3), a recognition event that is evolutionarily conserved, as evidenced by its presence across multiple animal species (171). Future elucidation of bile acid-receptor interactions is anticipated to emerge from comprehensive structural and functional analyses of SBAs.

## 5 Regulation of gut microbiota ecology by bile acids

The human gut microbiota, with an estimated cell count ratio of 1.3-2.3:1 relative to host cells, constitutes one of the most densely populated natural ecosystems identified to date (172). This intricate microbial community encompasses thousands of bacterial species, predominantly from the phyla Firmicutes and Bacteroidetes, which together represent approximately 90% to 99% of the total microbial population (173). The metabolic conversion of SBAs plays a pivotal role in maintaining gut microbiota homeostasis (**Table 2**). In patients suffering from cholestasis, there is a notable increase in intestinal bacterial overgrowth and bacterial translocation, both of which are significantly mitigated following SBA treatment (176). Conversely, administration of CA in rat models results in a marked proliferation of Firmicutes within the gut microbiota (177). A reduction in the bile acid pool is associated with the promotion of Gram-negative bacterial growth, which are frequently linked to pathogenicity (177). In contrast, an expanded bile acid pool supports the proliferation of Gram-positive bacteria within the Firmicutes phylum-many of which exhibit 7α-dehydroxylation activity and demonstrate increased tolerance to SBAs (178). Furthermore, SBAs contribute to colonization against *Clostridioides difficile* (179). Notably, restoration of SBAs production, through fecal microbiota transplantation (FMT) or supplementation with *Clostridium scindens*, has been shown to achieve cure rates of up to 95% in patients with recurrent *C. difficile* infection (CDI) (180, 181).

**Table 2:** Gut microbiota-mediated transformations of secondary bile acids.

Transformation type	Microbial enzyme(s)	Representative producer(s)	Products
bile acid hydrolase and conjugase	BSH (with aminoacyltransferase activity)	*Bacteroides*, *Lactobacillus*, *Bifidobacterium*	Unconjugated bile acids and microbially conjugated bile acids (MCBAs) (82, 83)
oxidation (C3, C7, C12)	3α/β-, 7α-, 12α-HSDHs	*Eggerthella lenta*, *Bacteroides* spp.	oxo-bile acids; UDCA (30, 66, 156)
C7-dehydroxylation	BaiE (7α-dehydratase)	*Clostridium scindens*, *Ruminococcaceae* spp.	DCA, LCA (74)
C3-dehydroxylation	Putative C3-dehydroxylase (not fully identified)	*Clostridium* spp.	5β-cholanic acid (77)
C12-dehydroxylation	Putative C12-dehydroxylase (not fully identified)	*Bacteroides* spp.	CDCA from CA; LCA from DCA (30, 79)
Epimerization or C12- oxidation	3β-, 7β-, 12β-HSDHs	*Clostridium absonum*, *Ruminococcus gnavus*	2-OxoCDCA; 12-oxoLCA; epiCA; epiDCA (30, 100)
Allo-secondary bile acid formation	BaiP/BaiJ + BaiA	*Clostridium* spp., *Bacteroidota*	alloDCA; alloLCA; isoDCA; isoLCA (174)
Bile acid sulfatase	Aryl sulfatases	*Peptococcus*, *Clostridium*, *Pseudomonas*	Unconjugtsed bile acids (175)

The composition of the gut microbiota is significantly influenced by its variable responsiveness to the bile acid pool *In vivo*, bile acids exhibit the antibacterial properties, while SBAs demonstrate potent inhibitory effects against specific microbial taxa (56, 182). These direct antimicrobial actions, deriving from its biological detergent properties, represent a critical physicochemical defense mechanism employed by the host (183). Elevated concentrations of bile salts disrupt microbial membrane lipids and dislodge integral membrane proteins, inducing transient solubilization. This result in leakage of cellular contents and rapid microbial cell death (184). Furthermore, bile acids directly impede bacterial proliferation by increasing membrane permeability, causing DNA and oxidative damage, and interfering with amino acid and ribosomal metabolism (56, 185, 186). The hydrophobicity of bile acids is a key factor determining their interaction with microbial membranes. Conjugated bile acids, which are fully ionized, predominantly localize within the outer leaflet of the lipid bilayer, whereas free bile acids can passively diffuse into cells, exerting a more pronounced inhibitory effect (187). Notably, free bile acids display greater antimicrobial potency compared to their conjugated forms (186). Consequently, Gram-negative bacteria, particularly members of the Bacteroidetes phylum, are susceptible to free bile acids, whereas Proteobacteria exhibit higher tolerance relative to Firmicutes and Actinobacteria (56, 186).

Responses to SBAs vary at the genus and species levels, especially among extensively studied probiotic genera such as *Bifidobacterium* and *Akkermansia* (56, 186). The outer membrane of Gram-negative bacteria serves as a protective barrier that impedes penetration of bile acids, and contains resistance-nodulation-cell division (RND) efflux pumps that reduce intracellular bile acid concentrations (188, 189). In contrast, the absence of an outer membrane in Gram-positive bacteria permits direct access of bile acids to the peptidoglycan layer and plasma membrane, facilitating solubilization. As a result, Gram-negative bacteria generally exhibit greater intrinsic tolerance to bile acids, as exemplified by the isolation of pathogens such as *Salmonella* and *Escherichia coli* from the gallbladder (190, 191).

The microbial metabolism of PBAs into SBAs results in the production of compounds exhibiting enhanced selective antimicrobial properties. In murine models, the incorporation of CA facilitates 7α-dehydroxylation and the accumulation of DCA, which promotes the proliferation of Firmicutes, particularly Clostridium cluster XIVa, while concurrently inhibiting the viability of Enterobacteriaceae (Ridlon et al., 2013). Analogously, short-term consumption of animal-based diets in humans rapidly elevates DCA levels, correlating with reduced the abundance of *Ruminococcus gnavus* (192). Furthermore, SBAs have been shown to suppress *Clostridium difficile*, a pathogen implicated in antibiotic-associated diarrhea (179).

Microbial transformation of bile acids also alters the composition of the bile acid pool, thereby selecting for bile-tolerant microbial communities. Pathogens like *Listeria monocytogenes* enhance their tolerance through the expression of BSH enzyme (193). Specific bile acid modifications also reshape microbial populations; oral administration of DCA in a murine model of carbon tetrachloride (CCl_4_)-induced liver injury increased the abundance of *Akkermansia muciniphila* while reducing populations of *Turicibacter* and *Lactobacillus* (194). Conversely, *Prevotella copri* disrupts bile acids metabolism in obese mice by elevating levels of PBAs and UDCA, concurrently inhibiting the proliferation of *Akkermansia* spp (195). Additionally, microbial cross-feeding mediated by bile acid metabolism has been observed. *Bacteroides uniformis* converts CA into 3-succholic acid (3-sucCA), which supports the growth of *Akkermansia muciniphila* and contributes to the maintenance of intestinal epithelial integrity (92). *Ruminococcus gnavus* has been identified as an efficient producer of iso-DCA, which transforms DCA into a less toxic derivative, which promotes the *in vitro* growth of *Bacteroides* in conjunction with another iso-DCA producer, *Eggerthia lenta* (73).

SBAs also indirectly influence the gut microbiota through activation of host bile acid receptors. Within the intestinal epithelium, the activation of the FXR induces the expression of genes involved in gut protection, thereby inhibiting bacterial overgrowth and mitigating mucosal injury (196). FXR-deficient mice exhibit increased bacterial loads in the ileum and compromised epithelial barrier function (196). Pharmacological activation of FXR in humans using obeticholic acid (OCA) suppresses endogenous bile acid synthesis and is associated with an increased abundance of Gram-positive bacterial strains, including *Streptococcus thermophilus*, *Lactobacillus casei*, *Lactobacillus paracasei*, *Bifidobacterium breve*, and *Lactococcus lactis*, suggesting that FXR activity modulates the small intestinal bile acid pool and consequently shapes microbiota composition (197). Supporting this notion, CA supplementation in Apc-Min^−/+^ mice leads to an increase in opportunistic pathogens such as *Prevotella* and *Desulfovibrio*, alongside a reduction in beneficial bacteria including *Ruminococcus*, *Lactobacillus*, and *Roseburia* (198). Similarly, diminished bile acid levels observed in cholestatic liver disease are linked to intestinal bacterial overgrowth (199). Collectively, these findings underscore the profound influence of the bile acid pool on gut microbial ecology through direct antimicrobial effects, selective pressures exerted by microbial bile acid transformations, and host receptor-mediated mechanisms. Therefore, modulation of bile acid composition constitutes a critical mechanism for regulating microbial community structure.

## 6 Immunomodulatory mechanisms of SBAs

Recent advancements in multi-omics technologies have illuminated the profound immunoregulatory capacity of SBAs, positioning them as key microbiota derived metabolites at the host-microbiota interface. Beginning with correlative observations, this section seeks to elucidate a causal link between alterations in specific bile acid concentrations and the fate and functional behavior of immune cells, with an emphasis on their implications in ADs.

### 6.1 Immunomodulatory mechanisms of SBAs

SBAs serve as critical signaling molecules that directly modulate immune cell activity through diverse molecular mechanisms. As comprehensively reviewed by Fiorucci et al. (200), bile acid receptors are widely expressed on immune cells, forming a complex communication network. *In vitro* and *in vivo* evidence delineates specific immunomodulatory pathways driven by distinct SBAs. isoDCA functions as an antagonist of FXR in dendritic cells (DCs), attenuating their immunostimulatory phenotype and indirectly promoting the expansion of colonic regulatory T cells (Tregs) without impinging on T helper 17 (Th17) cell differentiation (13). In parallel, isoalloLCA enhances the transcriptional activity of the *foxp3* locus, a master regulator of Treg development, by facilitating an active chromatin state at the conserved non-coding sequence 3 (CNS3) (201). This process is mechanistically dependent on the nuclear hormone receptor 4A1 (NR4A1), which binds to the *foxp3* locus to drive its activation (202).

The immunomodulatory repertoire of SBAs extends to the regulation of Th17 cells, a key pro-inflammatory subset. In murine models of dextran sulfate sodium (DSS)-induced colitis, both LCA and 3-oxo-LCA have been shown to restore colonic Retinoic acid receptor-related orphan receptor γ (RORγ)^+^ Treg populations via signaling through the VDR (203). Subsequent studies elucidated that 3-oxoLCA and iso-LCA directly inhibit the transcriptional activity of RORγt, the lineage-defining transcription factor for Th17 cells, thereby suppressing their differentiation (201, 204). isoalloLCA similarly curtails Th17 cell differentiation, an effect achieved without downregulating RORγt expression or compromising cellular viability, suggesting a distinct, non-toxic mode of action (201).

Beyond nuclear receptor-mediated transcriptional control, SBAs can modulate immune function through non-canonical pathways. A pivotal study by Cong et al. revealed that deoxycholic acid (DCA) directly interacts with the PMCA4 on CD8^+^ T cells (170). This interaction promotes calcium efflux, leading to the transcriptional downregulation of interferon-γ (IFN-γ), tumor necrosis factor-α (TNF-α), and granzyme B—thereby blunting CD8^+^ T cell cytotoxicity. Furthermore, the immunogenic properties of SBAs are profoundly shaped by gut microbial modifications. Notably, bacterial sulfation of isoalloLCA completely abrogates its capacity to induce Treg differentiation, highlighting how specific microbial transformations can critically determine the functional outcome of a bile acid metabolite (114).

Collectively, these findings illustrate that SBAs orchestrate immune responses through a multifaceted arsenal: nuclear receptor signaling, epigenetic regulation, and ion channel modulation. The functional consequence of any given SBA is inherently linked to its precise molecular structure, which is, in turn, a product of the gut microbial metabolome. This intricate interplay underscores the therapeutic potential of SBAs and their derivatives as sophisticated agents for immunomodulation.

### 6.2 Dysregulation of the SBA-immune axis in ADs

Aberrant immune activation lies at the core of ADs. While genetic susceptibility and environmental triggers are established contributors, compelling evidence now positions the gut microbiota and its metabolic output as a central player in the rising global incidence of ADs (205, 206). Dysbiosis, often coupled with increased intestinal permeability (leaky gut), facilitates the translocation of microbial components and metabolites into host systemic circulation, leading to persistent immune stimulation (207). Additional mechanisms, such as molecular mimicry and epitope sharing between microbial and host antigens, further cement the connection between intestinal microbes and the breakdown of self-tolerance (208, 209). Supporting this paradigm, experimental models of rheumatoid arthritis (RA) demonstrate that gut microbial alterations precede clinical disease onset, and interventions targeting the microbiome or intestinal barrier integrity can ameliorate symptoms, underscoring the microbiota’s causative role (210).

**Figure 4:**
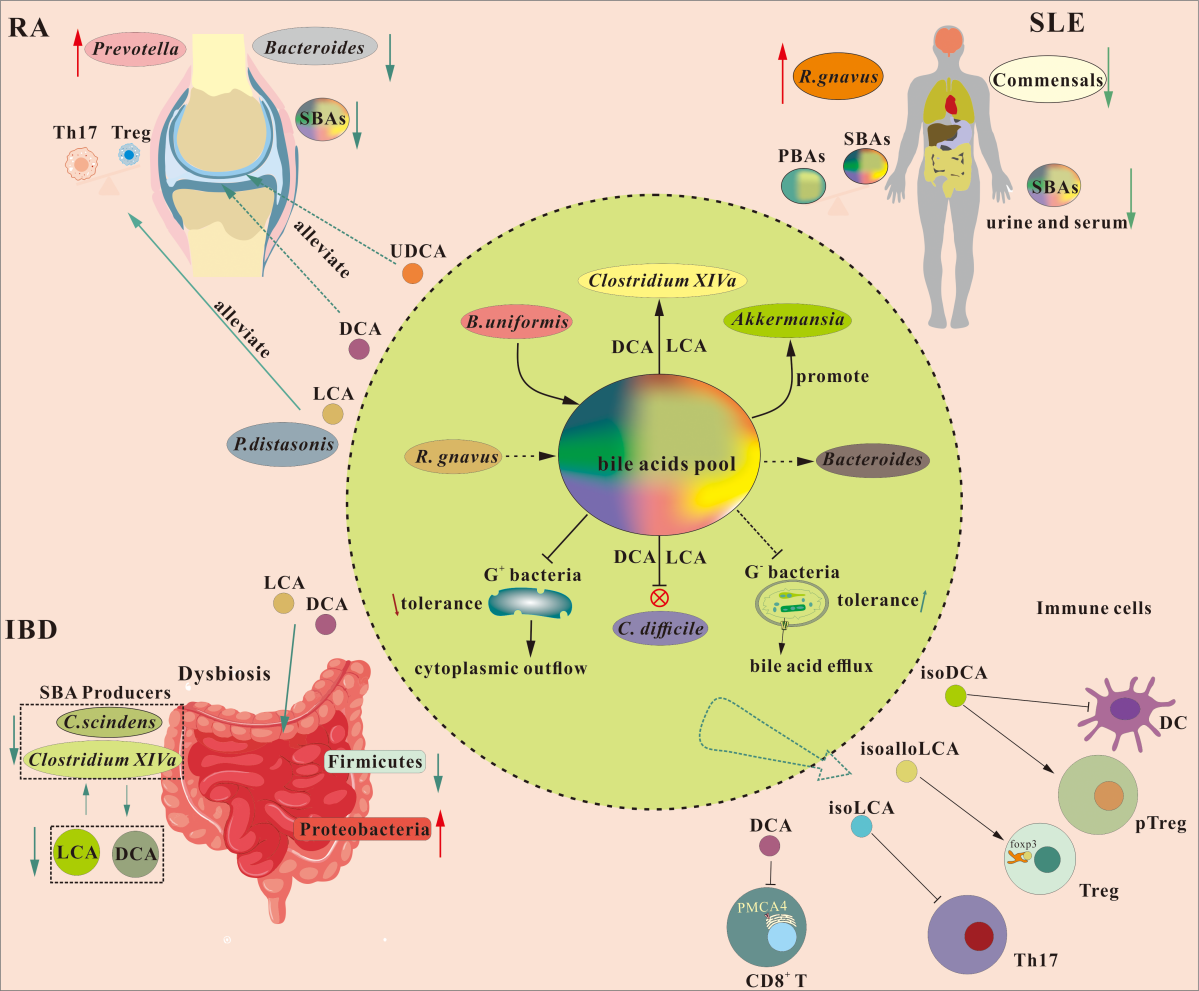
Regulation of intestinal flora ecology by the bile acid pool and its mediated immunomodulation in autoimmune disorders. Bile acids exert multifaceted roles in gut ecology and host immunity through direct antimicrobial effects, microbial community modulation, and immune regulation. Their detergent properties disrupt bacterial membranes and induce oxidative damage, with Gram-negative bacteria exhibiting higher tolerance via outer membrane barriers and efflux pumps. Gut microbes transform primary bile acids into secondary bile acids (SBAs), diversifying the bile acid pool and generating compounds with selective antimicrobial activity, which inhibit pathogens like Clostridium difficile. SBAs further shape microbial composition by promoting Firmicutes and Clostridium cluster XIVa while suppressing Enterobacteriaceae and *Ruminococcus gnavus*. Cross-feeding interactions also occur: *Bacteroides uniformis* produces 3−sucCA to support *Akkermansia*, whereas *R. gnavus* and *Eggerthia lenta* generate iso−DCA, which reduces toxicity and facilitates Bacteroides growth. In contrast, *Prevotella copri* metabolizes bile acids and reduces Akkermansia abundance. Beyond microbial ecology, SBAs directly regulate immune cells. Iso−DCA dampens dendritic cell activity and promotes colonic regulatory T cells. LCA and 3−oxo−LCA restore RORγ^+^ Tregs, while 3−oxoLCA and iso−LCA suppress Th17 differentiation by inhibiting RORγt. IsoalloLCA enhances chromatin accessibility at the Foxp3 locus, boosting Treg differentiation. DCA interacts with PMCA4, triggering Ca²^+^ efflux and inhibiting CD8^+^ T cell effector functions. Alterations in the SBA profile are closely linked to autoimmune diseases. In inflammatory bowel disease, dysbiosis correlates with diminished SBA levels; SBA supplementation alleviates inflammation and restores microbial balance. In rheumatoid arthritis, fecal SBAs are decreased, and SBA−sensitive *Prevotella copri* is enriched, potentially disrupting Th17/Treg homeostasis. Interventions with SBAs or SBA−producing bacteria ameliorate disease in experimental arthritis. Ursodeoxycholic acid also shows protective effects in osteoarthritis via the gut−joint axis. Systemic lupus erythematosus is associated with reduced SBAs, elevated primary bile acids, and expansion of *Ruminococcus gnavus*, highlighting the broad impact of bile acid metabolism on autoimmune pathogenesis.

Advances in next-generation sequencing (NGS) and integrated multi-omics approaches have enabled detailed mapping of gut microbial communities and their associated metabolomic signatures across various ADs, including inflammatory bowel disease (IBD), multiple sclerosis (MS), RA, type 1 diabetes (T1D), and systemic lupus erythematosus (SLE) (211–213). A consistent and prominent finding across these studies is the significant disruption of SBA profiles, which correlates strongly with clinical disease parameters. Building upon the fundamental immunomodulatory mechanisms outlined above, the following section synthesizes current knowledge on the specific alterations within the SBA pool and associated gut microbiota in ADs. We will explore how these disruptions compromise the SBA-immune network, examine the consequent immunomodulatory effects, and discuss emerging therapeutic strategies that aim to restore this delicate balance, with a focused analysis on IBD, RA, and SLE (**Figure 4**).

#### 6.2.1 Inflammatory bowel disease

IBD, encompassing ulcerative colitis (UC) and Crohn’s disease (CD), is characterized by chronic, relapsing inflammation of the gastrointestinal tract. Gut microbial dysbiosis is a well-established etiological factor driving its pathogenesis (214). Within this context, SBAs emerge as critical molecular links between microbial ecology and host inflammatory responses. A hallmark finding in IBD is a marked depletion of SBAs in the intestinal lumen, positioning the microbiota-bile acid axis as a focal point for mechanistic and therapeutic research (**Table 3**) (218, 219).

**Table 3:** Therapy targeting bile acids for IBD disease.

Subjects	Treatment	Samples and methods	Major findings
81 patients with active UC (215)	FMT (multi-donor fecal sample) or placebo; colonoscopic infusion, followed by enemas 5 days per week for 8 weeks	Fecal samples (metagenomic and metabolomic analysis) Colonic biopsy samples (16S rRNA gene and transcript sequencing)	In patients shows alleviation, *Eubacterium hallii* and *Roseburia inulivorans***↑** and SBAs**↑**. Metabolic pathways associated with SBAs**↑** in fecal samples from successful donors.
29 children with CD (216)	Infliximab infusion of 5 mg/kg at weeks 0, 2, and 6, followed by maintenance intravenous infusions every 8 weeks	Fecal samples (16S rRNA sequencing and UPLC-MS)	BSH producer (particularly Blautia and Collinsella)**↑**; conjugated BAs**↓**; free bile acids**↑**; 7α-dehydroxylating bacteria**↑**; the ratio of SBAs to PBAs**↑**.
Mice with DSS-induced chronic colitis (217)	Fucose gavage for 57 days	Ileal tissue lysates and colonic feces (16S rRNA sequencing and UPLC-MS)	Fucose ameliorates colitis through restoring the crosstalk between bile acid and gut microbiota.
Mice with DSS-induced acute colitis (218)	Daily gavage of UDCA, TUDCA, GUDCA (500 mg/kg) or placebo for 10 days	Fecal samples (16S rRNA sequencing and HPLC)	Reestablish the typical Firmicutes/Bacteroidetes ratio; Clostridium cluster XIVa, *Akkermansia muciniphila*, and Prevotellaceae**↑**; UDCA and LCA**↑**.
DSS, TNBS and CD45RBhi T cell transfer induced acute colitis in mice (219)	Enemas with 5 mg of DCA or LCA were administered on days 4, 6, and 8	Fecal samples (Metabolomics, microbiomics, metagenomics, and transcriptomics)	Significantly alleviating the symptoms of the colitis model depends on the expression of TGR5 in immune cells.
Single oral gavage GF IL-10 knockout mice with *Campylobacter jejuni* (10^9^ CFU/mouse) for 12 days (220)	Daily gavage with *in vitro* cultured aerobic microorganisms, anaerobic microorganisms, or conventionalized microbiota (10^8^ CFU/mouse) for 14 consecutive days; Daily gavage with 30 mg/kg DCA, LCA, or UDCA for 12 consecutive days.	Fecal samples (16S rDNA sequencing, multiparallel sequencing and transcriptomics)	Anaerobic flora alleviate inflammation by inhibiting mTOR through DCA.

The dysbiosis associated with IBD follows a recognizable pattern, consistently observed across patient cohorts and animal models. It is generally characterized by a reduced abundance of the phylum Firmicutes, a concomitant expansion of Proteobacteria, a loss of beneficial commensals, and an increase in potentially pathogenic taxa such as *Escherichia coli* (216, 221). This dysbiosis is often more severe in CD than in UC, reflected in lower overall microbial diversity and more pronounced compositional shifts. Specifically, CD patients show decreased abundances of Bacteroidales, Bacteroidaceae, Clostridiaceae, Ruminococcaceae, Lachnospiraceae, and *Clostridium scindens*, alongside an increase in Enterobacteriaceae, Pasteurellaceae, Veillonellaceae, and Fusobacteriaceae (222–224). In UC, reductions are noted in Ruminococcaceae, Lachnospiraceae, Clostridium cluster XIVa, *Butyricimonas*, *Eubacterium rectale*, *C. scindens*, and Roseburia, coupled with elevations in *Ruminococcus*, *Fusobacterium*, and *E. coli* (225, 226). Critically, the loss of SBA-producing bacteria like *C. scindens* and Clostridium cluster XIVa, directly undermines the generation of immunomodulatory SBAs.

This microbial shift translates into a functionally impaired bile acid metabolism. Metabolomic profiling confirms significant reductions in fecal SBAs, particularly DCA and LCA, which correlate with diminished expression of microbial bile acid transformation genes (223, 227). The functional consequence of this SBA deficiency is pro-inflammatory, while supplementation with SBAs has demonstrated efficacy in attenuating intestinal inflammation in murine models of colitis (219). In IL-10^-/-^ mice, increase in taurocholic acid (TCA) was associated with a significant proliferation of the pathogen *Bilophila wadsworthia*, leading to DC activation and subsequent Th1-mediated colitis, suggesting that interactions between bile acids and the gut microbiota may contribute to IBD pathogenesis (228). In an *in vitro* inflammation model of Caco-2 cells, DCA and LCA inhibit IL-1β-stimulated IL-8 secretion, whereas PBAs CA and CDCA do not, an effect nullified for LCA upon sulfation (229). Large-scale cohort studies have further substantiated that SBAs are markedly reduced in the feces of patients with IBD and show a positive correlation with fecal calprotectin levels, highlighting the potential involvement of SBAs depletion in IBD pathogenesis (223). Conversely, therapeutic supplementation with DCA or LCA in murine colitis models attenuates inflammation, reduces weight loss, improves histology, and decreases disease activity scores (219). Additional investigations also revealed that fecal metabolites of these SBAs (3-oxo-DCA, 3-oxo-LCA, alloLCA, isoalloLCA, and 3-oxo-UDCA) are inversely correlated with disease severity, with the reduction of 3-oxo-DCA exhibiting a particularly strong negative association. In TNBS- or DSS-induced murine models, 3-oxo-DCA exerted therapeutic effects by restoring the Firmicutes/Bacteroidetes ratio and activating the TGR5 (230). Furthermore, the balance between conjugated and unconjugated bile acids is considered critical in IBD progression. Impaired bile acid binding exacerbates disease severity in DSS-treated mice, and patients with CD display diminished diversity and abundance of intestinal *bsh* transcription. However, the abundance of *bsh* derived from *Ruminococcus gnavus* and *Enterococcus clostridioformis* is significantly elevated, suggesting that an altered microbial *bsh* profile, contributes to IBD pathogenesis (91).

These insights are informing novel clinical strategies. In pediatric IBD patients undergoing fecal microbiota transplantation (FMT), an unexpected decline in gut microbiota diversity to baseline low levels was observed six months post-treatment; nevertheless, SBAs concentrations remained consistent with those of the donor microbiota (231). This phenomenon, termed “metabolic persistence”, may attribute to specific strains introduced via FMT, or to metabolites that activate bile acid metabolic pathways within the microbial community, providing valuable insights for optimizing intervention strategies for IBD (232). Moreover, expression levels of gut microbial genes within *bai* operons serve as predictive biomarkers for differential therapeutic responses to anti-cytokine and anti-integrin therapies in IBD. Notably, patients exhibiting a *bai*-positive phenotype demonstrate higher remission rates following anti-cytokine therapy, associated with upregulation of microbial SBAs biosynthetic pathways. This predictive capability offers a promising approach to reduce treatment delays inherent in current empirical sequential therapeutic regimens (233).

#### 6.2.2 Rheumatoid arthritis

RA is a systemic autoimmune disease primarily affecting the joints, leading to progressive functional impairment and elevated mortality rates (234). Accumulating evidence underscores a significant role for gut microbial dysbiosis in its pathogenesis, as observed in both RA patients and experimental murine models (210, 235).

A distinctive feature of this dysbiosis is the enrichment of *Prevotella copri*, a species sensitive to SBAs (236). The abundance of Prevotella species, particularly *P. copri*, is elevated in the gut microbiota of individuals with preclinical RA (237). Experimental evidence from collagen-induced arthritis (CIA) mouse models indicates that intestinal colonization by *Prevotella* spp. exacerbates joint inflammation (238). Newly diagnosed RA patients also show increased *P.copri* levels alongside a reduction in *Bacteroides fragilis*, compared to individuals with chronic RA, psoriatic arthritis (PsA), and healthy subjects (239). These microbial shifts correlate with alterations in the bile acid pool. While serum bile acid concentrations may not show gross changes in RA, studies in related conditions like psoriatic arthritis (PsA) confirm a link between SBAs and disease activity (240). Notably, fecal concentrations of key immunomodulatory SBAs, DCA, LCA, 3-oxoLCA, and isoLCA are significantly reduced in both RA patients and CIA mouse models (241). Restoring this deficit, either by colonizing mice with the SBA-producing bacterium *Parabacteroides distasonis* (possesses 3β-HSDH and 3α-HSDH) or by directly supplementing with LCA, 3-oxoLCA, or isoLCA, ameliorates arthritis severity in CIA models. This therapeutic effect is mediated by the rebalancing of Th17 and Treg differentiation (241, 242).

The broader relevance of bile acids in joint homeostasis is further illustrated in osteoarthritis (OA). OA patients exhibit reduced gut microbial diversity and a specific decrease in *Clostridium bolteae*, a producer of UDCA, leading to lower UDCA levels. Oral administration of UDCA or *C.bolteae* has been shown to mitigate OA pathology in murine models by inhibiting FXR in intestinal L cells, thereby enhancing the secretion of glucagon-like peptide-1 (GLP-1). Circulating GLP-1 subsequently targets joint tissues, promoting cartilage synthesis in chondrocytes and synovial cells via a specific receptor (243). These findings collectively delineate a gut microbiota–bile acid–joint axis, through which microbial metabolites systemically influence articular health and autoimmune processes.

#### 6.2.3 Systemic lupus erythematosus

SLE is characterized by a clinical course marked by recurrent episodes of remission and exacerbation, commonly presenting with symptoms such as arthritis, cutaneous lesions, and involvement of systemic organs (244). While overt gastrointestinal symptoms are often mild and may be treatment-related, subclinical gut dysbiosis is increasingly recognized as a contributing factor (11). A serious complication, lupus nephritis, develops in over 50% of patients, with approximately 20% progressing to end-stage renal disease (245).

The gut dysbiosis in SLE parallels patterns seen in other extra-intestinal autoimmune diseases. Disease activity is frequently associated with an increased abundance of the mucolytic bacterium *Ruminococcus gnavus* (246–248). Metabolically, this dysbiosis is reflected in a disturbed bile acid profile. Comparative analyses reveal that SLE patients have elevated levels of PBAs and concomitant reductions in SBAs in both fecal and urine samples (213). A non-targeted metabolomics study of Colombian SLE cohorts, including patients with lupus nephritis, identified the primary bile acid synthesis pathway as the most significantly altered metabolic pathway, underscoring the systemic disruption of bile acid homeostasis in this disease (249). These observations suggest that an altered bile acid milieu, likely stemming from gut microbial imbalance, may contribute to the loss of immune tolerance and inflammatory cascade characteristic of SLE.

### 6.3 Targeting SBAs and their receptors: therapeutic horizons

The modulation of gut microbial ecology to enhance immunomodulatory SBAs production presents a strategic avenue for ADs prevention. For high-risk individuals, interventions aimed at restoring a balanced SBA profile, via defined microbial consortia or targeted SBA supplementation, can promote an anti-inflammatory, tolerogenic milieu (250). However, the hepatotoxicity and context-dependent efficacy of natural SBAs necessitate rigorous preclinical evaluation to establish safe therapeutic windows. Complementarily, synthetic bile acid receptor agonists offer a direct pharmacological strategy to activate beneficial signaling pathways, bypassing microbial constraints. While promising for systemic immunomodulation, this approach carries risks of metabolic side effects, receptor desensitization, and disruption of bile acid homeostasis, mandating careful dose optimization and monitoring (251).

In summary, therapeutic modulation of the bile acid axis holds considerable promise for both the prevention and treatment of autoimmune diseases. Next-generation approaches, including engineered live biotherapeutics and precision metabolites, hold potential for localized signal delivery with minimized systemic exposure. To successfully translate these compelling mechanistic insights into safe and effective clinical applications, conducting rigorous long-term safety studies and deepening our understanding of the complex interplay between diet, host genetics, and the gut microbiome will be of paramount importance.

## 7 Outlook

The significance of SBAs, as pivotal metabolites produced by the gut microbiota, has been the subject of extensive investigation over several decades, particularly concerning their influence on the gut ecosystem. Recent technological advancements in high-throughput sequencing, small molecule analytics, and artificial intelligence have markedly enhanced our understanding of the diversity and functional roles of bile acids in microbiota-host communication. Nonetheless, the specific contributions of SBAs to microbial homeostasis and immune regulation within the host, as well as their precise interactions with immune cell-associated receptors, remain insufficiently characterized. Emerging studies on novel microbiota-derived bile acids, including amino acid-conjugated and isomerized variants, highlight a complex and multifaceted dialogue between the gut microbiome and the host immune system. Future research endeavors should focus on delineating the tissue-specific distribution of SBAs, identifying novel structural variants, and elucidating the detailed conformational interactions between SBAs and their cognate receptors. Moreover, given the central role of gut microbiota in SBAs biosynthesis, a deeper understanding of the microbial genetic determinants governing bile acid metabolism is essential for comprehending the pathways involved in their transformation and modification. Utilizing established gnotobiotic mouse models, genetically engineered bacterial strains with customized bile acid-metabolizing enzymes, and conditional knockout animal models will enable researchers to further unravel the complex mechanisms underlying bile acid-host interactions, thereby offering novel insights into the gut-microbiota-organ axis.
